# Simultaneous Surgical Management of Acute Tibial Shaft Fracture and Post-traumatic Ankle Arthritis

**DOI:** 10.7759/cureus.33025

**Published:** 2022-12-27

**Authors:** Abdullah Alzahrani, Ali Alshehri, Khalid Alsheikh, Faisal Alzahrani, Rand A Alshaya, Ibrahim Ababtain

**Affiliations:** 1 Department of Orthopedic Surgery, King Abdulaziz Medical City, Riyadh, SAU; 2 College of Medicine, King Saud Bin Abdulaziz University for Health Sciences, Riyadh, SAU; 3 Department of Orthopedic Surgery, Prince Sultan Military Medical City, Riyadh, SAU

**Keywords:** case-report, retrograde intramedullary femoral nail, post-traumatic ankle arthritis, tibial shaft fracture, tibiotalocalcaneal arthrodesis

## Abstract

The simultaneous management of tibial shaft fractures and post-traumatic ankle arthritis with ankle pain as the chief complaint can be challenging. Herein, we present a case managed with closed reduction, internal fixation, and tibiotalocalcaneal arthrodesis using a retrograde femoral nail. The patient was able to actively ambulate with full weight bearing and no pain approximately three months after the procedure. Patient consent for this case report was obtained.

## Introduction

Tibial shaft fractures are severe injuries that could result in complications such as acute compartment syndrome, deep vein thrombosis, infection, loss of alignment, nerve injury, nonunion, reduced mobility, and permanent disability [1-5]. The gold standard surgical procedure for tibial shaft fractures is reamed intramedullary nailing [1,6]. However, a search in the literature did not yield results regarding the management of acute tibial shaft fracture combined with post-traumatic ankle arthritis with ankle pain as the main complaint. Post-traumatic ankle arthritis develops secondary to joint trauma and is usually managed by either total ankle replacement or ankle arthrodesis [7].

Managing a tibial shaft fracture combined with post-traumatic ankle arthritis with ankle pain as the chief complaint can be challenging; a thorough search of the previous literature failed to reproduce any results pertaining to this combination. One possible solution is to simultaneously perform closed reduction and internal fixation (CRIF) and tibiotalocalcaneal arthrodesis (TTCA) using a retrograde femoral nail to achieve a stable and pain-free function of the lower limb. The instrument chosen, although it violates the subtalar joint, fixates the tibial fracture, manages severe ankle arthritis, and, if done correctly, establishes and maintains proper lower-limb alignment. To the best of our knowledge, the previous literature does not describe a case using the same technique for the same purpose.

This case report demonstrates the surgical technique and outcomes of CRIF and TTCA using a retrograde femoral nail in a patient with a right spiral tibial shaft fracture and severe post-traumatic ankle arthritis.

## Case presentation

A 71-year-old male patient with a history of type II diabetes mellitus, hypertension, and dyslipidemia presented to the emergency department (ED) with a chief complaint of pain in his right leg and ankle after falling out of bed and twisting his ankle. The leg pain started after the injury; however, he suffered from severe ankle pain two years before the injury, which prevented him from ambulating outside his house. This long-lasting ankle pain started after a previous right ankle fracture, which was managed by open reduction and internal fixation (ORIF) three years before this visit. He was following with podiatry in a peripheral town and was only offered boots, to which he did not respond very well. 

Physical examination revealed that he was on a splint from the emergency medical services. His right ankle was swollen and edematous, with tenderness over the proximal lateral and distal medial leg and ankle tenderness, indicating the presence of an injury and the significance of his ankle arthritis. Furthermore, the posterior tibial and dorsalis pedis arteries were not palpable, but they were triphasic on Doppler ultrasound. Moreover, the patient was able to move his foot and toes, and the sensation was intact.

**Figure 1:**
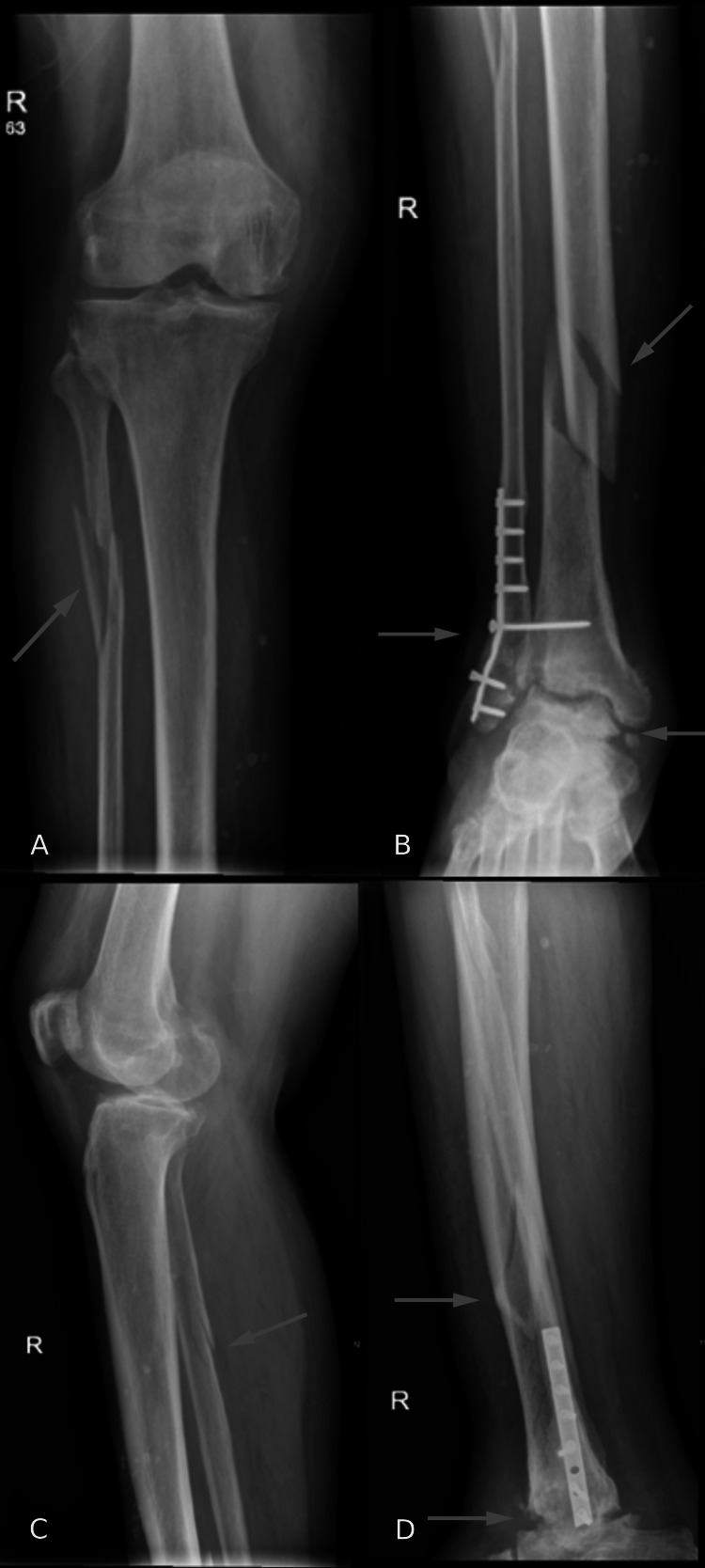
(A, B) Anteroposterior and (C, D) lateral X-rays of the right tibia and fibula after the injury

**Figure 2:**
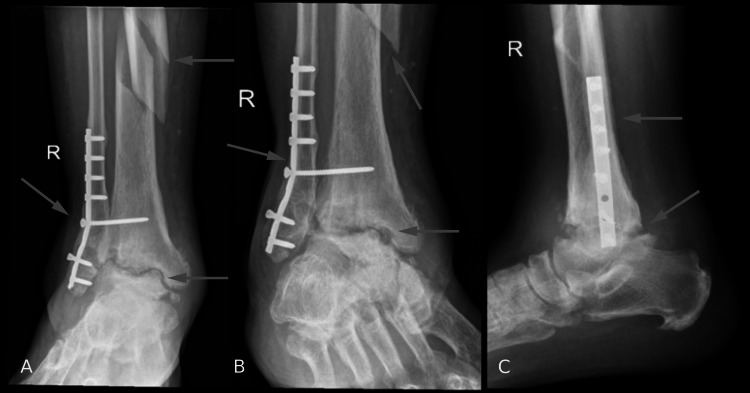
(A) Anteroposterior, (B) oblique, and (C) lateral X-rays of the ankle joint after the injury

Radiographs of the patient’s right tibia, fibula, and ankle were taken by the ED and are shown in Figures 1, 2. Radiographs of the tibia and fibula showed a comminuted and mildly displaced proximal fibular fracture and tibial spiral fracture with medial displacement of the distal shaft of the tibia. Ankle radiography showed post-ORIF status with plates and screws transfixing distal fibular fracture with pulled-out screws. Moreover, radiography showed severe tibiotalar post-traumatic arthritis.

**Figure 3:**
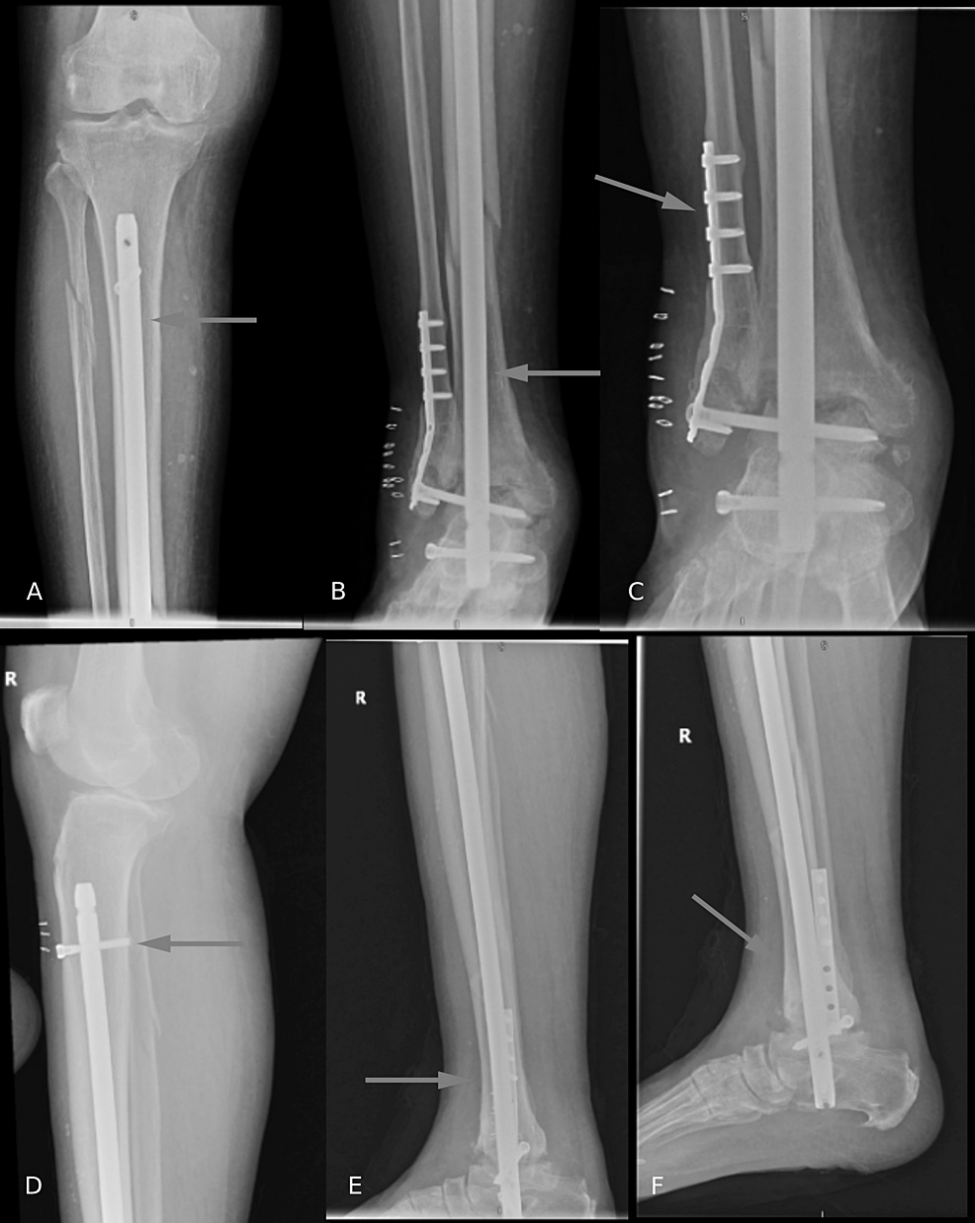
X-rays after the surgery. (A, B) Anteroposterior view of the right tibia and fibula, (C) anteroposterior X-ray of the ankle joint, (D, E) lateral views of the leg, (F) lateral view of the ankle

The patient was admitted to our hospital with an above-knee backslab. Subsequently, preoperative clearance was performed. An infection workup was conducted, and it was negative. He underwent closed reduction of the right tibia and ankle with retrograde nailing through the calcaneus using a retrograde femoral nail to fuse the ankle and fixate the fracture (Figure 3). He tolerated the procedure without complications and was referred for physiotherapy for non-weight-bearing on the right side, no ankle range of motion (ROM), and full-knee ROM.

**Figure 4:**
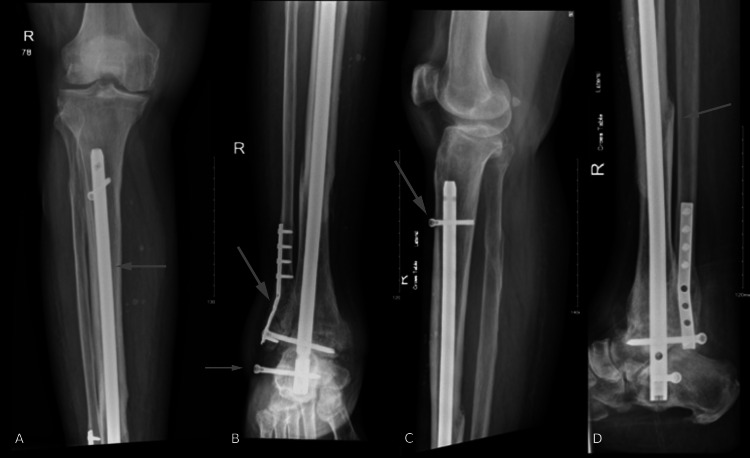
(A, B) Anteroposterior and (C, D) lateral X-rays of the right tibia and fibula on the latest follow-up

**Figure 5:**
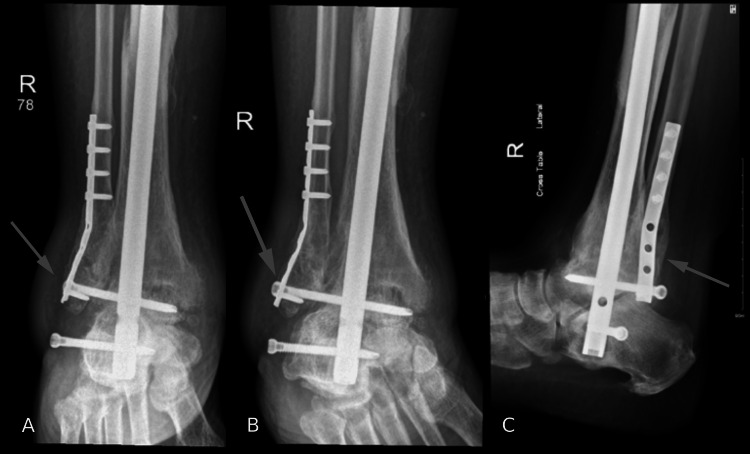
(A) Anteroposterior, (B) oblique, and (C) lateral X-rays of the right ankle joint on the latest follow-up

He started mobilizing with walking frames three days after the procedure and was discharged the next day. The patient was discharged with regular follow-ups. At the three-month follow-up, the bony union was achieved with a resulting moderate pullout of the lateral to medial screw at the calcaneus level that was asymptomatic. The medical team has discussed the possibility of follow-up removal surgery, but the patient is against it as he was not bothered by it, even though it can be felt under the skin. Full weight bearing, as tolerated, was started three months after the surgery. Five and a half months after the surgery, he was using a cane; however, he could walk without it. Around nine months after the procedure, the patient was actively mobile with full weight bearing. At the latest follow-up, 17 months after the surgery, the patient was doing well with no complaints or weight-bearing restrictions on the right ankle. In addition, the patient no longer complained of ankle pain. Radiographs of the tibia, fibula, and ankle at the latest follow-up are shown in Figures 4, 5, respectively.

Surgical technique

The patient was placed in a supine position, with his ankle hanging from the edge of the bed. Usually, positioning the patient in a prone position makes the procedure easier. However, due to the patient’s weight and comorbidities, the anesthesia team preferred to do it under regional anesthesia with the patient supine to monitor him. It was done in the supine position with hip and knee flexion. An incision was made on the fibular side of the syndesmotic screw under radiographic guidance. The syndesmotic and fibular pulled-out locking screws from the previous fixation were removed. Subsequently, a plantar-based incision in line with the talus and tibia was made under radiographic guidance. A guidewire was inserted under radiographic guidance through the calcaneus, talus, and tibia.

Subsequently, a proximal reamer was used to open the pathway for the ball-tipped guidewire, which was introduced up to the proximal tibia. Next, the reaming was started with 9 to 13 using a flexible power reamer. The length measurements were then taken at 400 mm, and the nail was inserted (retrograde femoral nail size [11.5 × 400 mm]). Distal screws were inserted through the calcaneus and the talus, followed by the insertion of a proximal screw into the nail.

Finally, radiographs of the fracture and ankle joint were taken, which showed satisfactory alignment. Forward hammering after the distal interlocking screw closed the fracture gap first definitely, then closed the small gap at the joint level eventually, which gave the final compression at both sites.

## Discussion

We decided to do a simultaneous tibial CRIF and TTCA because the patient presented with ankle pain as the main complaint. Furthermore, he stated the importance of dealing with his ankle pain before dealing with his leg. Therefore, we seized the opportunity to treat both using a single construct once and for all.

Most tibial shaft fractures occur in young and active patients and result from high-energy trauma, such as motor vehicle accidents or falls from height [1]. However, the second peak of incidence is seen among elderly patients whose injuries most likely result from a simple fall [1]. These fractures are generally managed with CRIF using an intramedullary nailing technique [1]. Reaming before nailing lowers the incidence of implant failure [6]. Therefore, reaming was done in our case.

We preferred using a femoral nail because femoral nails provide a comprehensive size range in terms of length and diameter and have less inherent angulation. Moreover, the use of femoral nails for TTCA has been well documented in the literature with excellent results [7-10]. In a prospective study [9], TTCA was performed using a retrograde femoral nail among 29 patients and showed that the mean American Orthopaedic Foot and Ankle Society Ankle-Hindfoot Score increased from 46 to 71 after surgery. Furthermore, using the 36-Item Short Form Health Survey Questionnaire, before the surgery, 73% of patients reported that their physical and mental health interfered with social activities some of the time. However, after the surgery, 86% of them rated their general health as excellent or good, 79% of patients reported less or no pain in the ankle and subtalar joints, and 82% had some improvements in quality of life after the surgery. Nevertheless, we could not find any previous research demonstrating the outcomes of using a femoral nail to fix a tibial shaft fracture and ankle arthrodesis.

We performed the surgery using a retrograde femoral nail because it does not invade the proximal tibia, which might compromise its strength. Moreover, according to a prospective study by Goebel et al. [10], the retrograde approach results in a higher rate of bone consolidation. The study included 20 patients who underwent TTCA using the retrograde approach and 20 who underwent TTCA using an antegrade approach. The results showed that the rate of bone consolidation was 95% in the retrograde group and 85% in the anterograde group. However, both groups demonstrated improvement in pain and quality of life.

Some limitations of this technique are that it is technically demanding due to the patient's position and ensuring the proper alignment and compression of the fracture and the joint. Furthermore, reaming would be challenging if the intramedullary canal diameter is less than 7 mm [11]. Moreover, this technique would not be possible if the intramedullary canal is deformed. On the other hand, one of the advantages of this technique is that the internal fixation can be dynamized. Furthermore, this technique in our patient resulted in significant pain improvement with the maintenance of appropriate hindfoot and midfoot alignment.

The patient's ankle pain improved significantly after surgery, and the tibial fracture healed adequately. In addition, the patient could fully bear weight on his ankle without any complaints at the three-month follow-up.

## Conclusions

Using a retrograde femoral nail for simultaneous tibial shaft fracture CRIF and ankle arthrodesis has proven successful in our case. The patient was able to walk three months after the surgery, did not report any pain, and was satisfied with the results.
